# Microenvironmental Targets in Sarcoma

**DOI:** 10.3389/fonc.2015.00248

**Published:** 2015-11-04

**Authors:** Monika Ehnman, Olle Larsson

**Affiliations:** ^1^Department of Oncology-Pathology, Cancer Center Karolinska, Karolinska Institutet, Karolinska University Hospital, Stockholm, Sweden

**Keywords:** sarcoma, microenvironment, angiogenesis, stroma, targeted therapy, VEGF, PDGF, KIT

## Abstract

Sarcomas are rare malignant tumors affecting all age groups. They are typically classified according to their resemblance to corresponding normal tissue. Their heterogeneous features, for example, in terms of disease-driving genetic aberrations and body location, complicate both disease classification and development of novel treatment regimens. Many years of failure of improved patient outcome in clinical trials has led to the conclusion that novel targeted therapies are likely needed in combination with current multimodality regimens. Sarcomas have not, in contrast to the common carcinomas, been the subject of larger systematic studies on how tumor behavior relates to characteristics of the tumor microenvironment. There is consequently an urgent need for identifying suitable molecular targets, not only in tumor cells but also in the tumor microenvironment. This review discusses preclinical and clinical data about potential molecular targets in sarcomas. Studies on targeted therapies involving the tumor microenvironment are prioritized. A greater understanding of the biological context is expected to facilitate more successful design of future clinical trials in sarcoma.

## Introduction

Sarcomas represent rare malignant tumors of mesenchymal origin with more than 70 different entities (1). Multimodal treatment generally includes local control by surgery and/or radiation and systemic control by chemotherapy. Specific treatment protocols depend on clinical parameters, including stage classification, histological grade, tumor site, and subtype. Most of the sarcoma subtypes are relatively resistant to chemotherapy.

Diagnosis and treatment regimens should be carried out in a multidiscipline manner. It is therefore highly recommended that patient care is centralized to a sarcoma reference center immediately after the initial detection of a suspected sarcoma. The European Sarcoma Network Working Group has developed clinical practice guidelines for diagnosis, treatment, and follow-up in bone sarcomas (2), soft tissue, and visceral sarcomas (3), and gastrointestinal stromal tumors (GIST) (4).

Patient outcome is influenced by many parameters. Local tumor recurrence and metastatic spread at diagnosis are associated with a poor prognosis. Curative treatment options are limited in these patient groups. Better treatment options are also needed to reduce long-term complications for patients in remission. This is particularly important to consider in pediatric patients where established treatments of today are known to be associated with, e.g., reduced fertility later in life.

Targeted therapies are currently emerging as a promising complementary or alternative treatment in several pathological entities including GIST (5, 6). Attempts have also been made to identify patient subgroups that are likely to benefit from this novel type of treatment. Given the mechanistic nature of targeted therapies, a molecular target essential for disease progression of each individual tumor typically needs to be identified. Detailed knowledge on sarcoma biology is therefore important for clinical progress.

## Subtype Classification in Sarcoma

Subtype classification in sarcoma is today guided by the 2013 World Health Organization (WHO) classification system (7). Dominating soft tissue sarcoma (STS) subtypes include undifferentiated pleomorphic sarcoma (UPS) and liposarcoma in adults and rhabdomyosarcoma in children. Also GIST is now a dominating subtype in adults after the inclusion in the soft tissue section. Common adult bone sarcomas include osteosarcoma and chondrosarcoma. Osteosarcoma is also a dominating subtype in children together with Ewing sarcoma. Examples of sarcoma subtypes according to their differentiation status are presented in Table 1.

**Table 1:** **Schematic overview of sarcomas of soft and bone tissue**.

**Differentiation of soft tissue sarcomas^a^**	**Examples**
Adipocytic tumors	Dedifferentiated liposarcoma
Fibroblastic/myofibroblastic tumors	Fibrosarcoma
Smooth muscle tumors	Leiomyosarcoma
Pericytic (perivascular) tumors	Malignant glomus tumor
Skeletal muscle tumors	Embryonal rhabdomyosarcoma
Vascular tumors	Angiosarcoma
Chondro-osseous tumors	Extraskeletal chondrosarcoma
Gastrointestinal tumors	Gastrointestinal stromal tumor (GIST)
Nerve sheath tumors	Malignant peripheral nerve sheath tumor
Tumors of uncertain differentiation	Synovial sarcoma
Undifferentiated/unclassified sarcomas	Undifferentiated pleomorphic sarcoma (UPS)
**Differentiation of bone sarcomas^b^**	**Examples**
Chondrogenic tumors	Chondrosarcoma grade ll–lll
Osteogenic tumors	Conventional high-grade osteosarcoma
Fibrogenic tumors	Fibrosarcoma
Osteoclastic giant cell rich tumors	Malignancy in giant cell tumor of bone
Notochordal tumors	Chordoma
Vascular tumors	Angiosarcoma
Myogenic tumors	Leiomyosarcoma
Lipogenic tumors	Liposarcoma
Miscellaneous tumors	Ewing sarcoma

*Modified from the present WHO classification of tumors (8*).

*^a^Dominating histotypes: UPS, liposarcoma (adults), rhabdomyosarcoma (children, young adults). Also GIST is now a dominating histotype in adults after the inclusion in the soft tissue section*.

*^b^Dominating histotypes: Osteosarcoma, chondrosarcoma (adults), osteosarcoma, Ewing sarcoma (children, young adults)*.

Some of the entities are classified with the help of cytogenetics, or molecular genetics, searching for characteristic genetic aberrations including pathognomonic fusion genes. A large group of sarcomas is however currently being classified according to exclusion criteria when a more precise categorization fails. Many of these tumors are referred to as UPS, or earlier, malignant fibrous histiocytoma. It is reasonable to assume that the classification of tumors belonging to this latter group will further improve with an increased molecular understanding.

Inconsistent classification of sarcomas has complicated registry-based studies, both over time and between countries. With increased knowledge about sarcoma biology, subtype classification is likely to rely more on molecular hallmarks in combination with observations made by conventional histology and selected imaging assessments (9). This will allow more stringent analyses in defined patient groups and ultimately improve patient outcome.

## Genetic Aberrations of Common Sarcoma Subtypes

We are only in the beginning of understanding the genetic aberrations involved in sarcoma development and progression. It has been recognized that genetic aberrations often lead to activation of tyrosine kinase growth factor receptors (10). However, distinct oncogenic drivers at the molecular level are often only detected in subsets of a defined sarcoma entity. The histology that traditionally aids sarcoma subtype classification is therefore, to our understanding today, only partially linked to the molecular profile of the tumor.

Specific genetic aberrations are commonly found in the class of sarcomas with a simple, or close to simple, karyotype. The confirmed presence of disease-driving, activating mutations in KIT or PDGFRα in GIST can today, e.g., be used to predict tumor recurrence and identify patients who are likely to benefit from adjuvant therapy (11).

Ewing sarcoma and alveolar rhabdomyosarcoma are both examples of translocation-associated sarcomas. The most common translocations involve transcription factors that actively dysregulate gene expression. EWS/FLI in Ewing sarcoma has been shown to upregulate the PDGFR ligand PDGF-C (12). The oncogenic fusion gene PAX3-FOXO1, found in the majority of all alveolar rhabdomyosarcomas, has been linked to poorer patient outcome (13, 14). Other examples of sarcomas with specific genetic aberrations involving chromosomal translocations are synovial sarcoma, clear cell sarcoma of soft tissue, myxoid, and round cell liposarcoma.

Many sarcomas are, however, known to have a more complex karyotype. Pleomorphic undifferentiated sarcoma, pleomorphic and dedifferentiated liposarcoma, leiomyosarcoma, pleomorphic rhabdomyosarcoma, osteosarcoma, and chondrosarcoma all belong to this other main class of sarcomas (10). The disease-driving mechanisms of these tumors are likely to be related to defects in the cell cycle checkpoints and the genetic instability as such.

## The Sarcoma Tumor Microenvironment

The microenvironment of tumors is composed of multiple stromal cell types and extracellular matrix proteins in addition to the cancer cells. The stromal compartment is typically involved in structural and functional support of tumor growth and coevolves together with the tumor cells in a unique manner depending on tumor type and tumor stage. It had already been described in 1960, how a primary tumor of sarcoma can evade the anticancer immune response by establishing an immune-privileged microenvironment (15, 16). In the 1970s, Juda Folkman proposed that angiogenesis is essential for solid tumor growth (17).

The importance of the tumor microenvironment for metastatic growth was addressed in the seed and soil theory (18). This theory describes the metastatic-prone tumor cell as “the seed” and the preferred metastatic site as “the soil.” Another complementary theory is the anatomical-mechanical hypothesis (19) suggesting that tumor cells are rather passively shed from the primary tumor by lymphatic drainage and the vascular system. This would then result in metastatic growth at anatomically accessible sites defined by the location of the primary tumor.

Both theories on the metastatic process are today considered applicable in a tumor-specific context (20, 21). The importance of tumor cell intravasation into the vascular system has particularly been addressed in sarcoma, where vascular invasion, as defined by the presence of tumor cells within any space having an endothelial lining, has been identified as a prognostic factor for metastasis (22). In the study by Engellau et al., vascular invasion was detected in 50 of 140 STS and was shown to closely associate with necrosis and malignancy grade.

### Blood Vessel-Associated Cells, Angiogenesis, and Prognostic Biomarkers

Blood vessels in tumors are composed of endothelial cells and various amounts of supportive mural cells including pericytes and vascular smooth muscle cells. The most commonly used markers for endothelial cells are CD31, CD34, VEGFR2, factor VIII, vWF, and endoglin. Podocalyxin was used as a vessel marker in experimental rhabdomyosarcoma to confirm the antiangiogenic response to sorafenib (23). CD31 is often considered to be a pan-endothelial marker, whereas endoglin is expressed by activated endothelium. Endoglin is also expressed by tumor cells in Ewing sarcoma, where its expression has been shown to correlate with worse patient survival (24).

In 1999, Tomlinson et al. concluded that the pattern of angiogenesis is different between sarcomas and carcinomas (25). Their study showed that the capillaries in carcinomas are clustered in bursts within the tumor stroma and that the microvessel density in these bursts can be used as a prognostic factor. By contrast, microvessel density in sarcomas was shown to have a more homogeneous appearance. A more recent study confirmed this pattern of angiogenesis showing that hot spots of angiogenesis were diffuse in high-grade STS and only present in 33% of the investigated specimens (26).

High microvessel density, as assessed by CD31 staining, has been shown to correlate with, e.g., high VEGF expression, tumor size ≥5 cm and high tumor grade in GIST (27). Microvessel density in other sarcomas has been evaluated, but occasionally also questioned in terms of inconsistently used methodologies and prognostic relevance (26, 28–32). Most studies on angiogenesis have chosen to focus on VEGF detection rather than microvessel density (33, 34). Both VEGF expression and circulating VEGF levels are of suggested clinical relevance in sarcomas. VEGF and additional markers of angiogenesis in sarcomas have been reviewed elsewhere (33, 35, 36).

### Other Infiltrating Stromal Cell Types in Sarcoma

The importance or prognostic relevance of infiltrating stromal cell types in sarcomas has not been extensively characterized. Some functions have been associated, directly or indirectly, with angiogenesis or vasculogenesis. Recruited CD34+ bone marrow stem cells have been shown to contribute to the growing tumor vasculature in response to VEGF in experimental Ewing sarcoma (37). M2-like, CD163+ CD14+ macrophages have been described in naïve ASPS. These tumor-associated macrophages of myeloid origin are believed to promote tumor progression and possibly VEGF-mediated vasculogenesis (38). In contrast, M1 macrophages are typically tumor preventing and respond to interferon-γ (39). Cavnar et al. recently suggested that M1/M2 macrophage polarization is linked to oncogene activity in a mouse model of spontaneous GIST as well as in human GIST (40).

Several studies on patient material have brought attention to lymphocytes. Selected examples include a study describing high prevalence of tumor-infiltrating lymphocytes in STS (41). Others have shown that tumor-infiltrating CD3+ lymphocytes in GIST correlated with improved progression-free survival (42). Infiltration of CD8+ lymphocytes in Ewing sarcoma correlated with improved survival (43). Expression of immune checkpoint molecules, such as the T cell-suppressive receptor PD-1, has been correlated with disease progression in, e.g., osteosarcoma (44). CD20+ lymphocytes have been associated with improved survival in a study on STS (45).

Sarcomas are often considered to be one-compartment tumors harboring limited activity from infiltrating fibroblast-like cells (25). PDGFR+ stromal cells have been described in human rhabdomyosarcoma, where their presence showed a clinical association with subtype and metastasis (23). Frequently detected stromal components (osteoid, cartilage, or collagen) are however not likely attributed to infiltrating fibroblasts in the majority of sarcomas, but rather the sarcoma cells and/or the tissue-specific cells, themselves (46). Some investigators have suggested that stromal fibrosis/hyalinization is a specific pattern associated with a non-viable tumor following treatment in STS (47).

## Therapeutic Targeting of the Sarcoma Microenvironment

**Figure 1:**
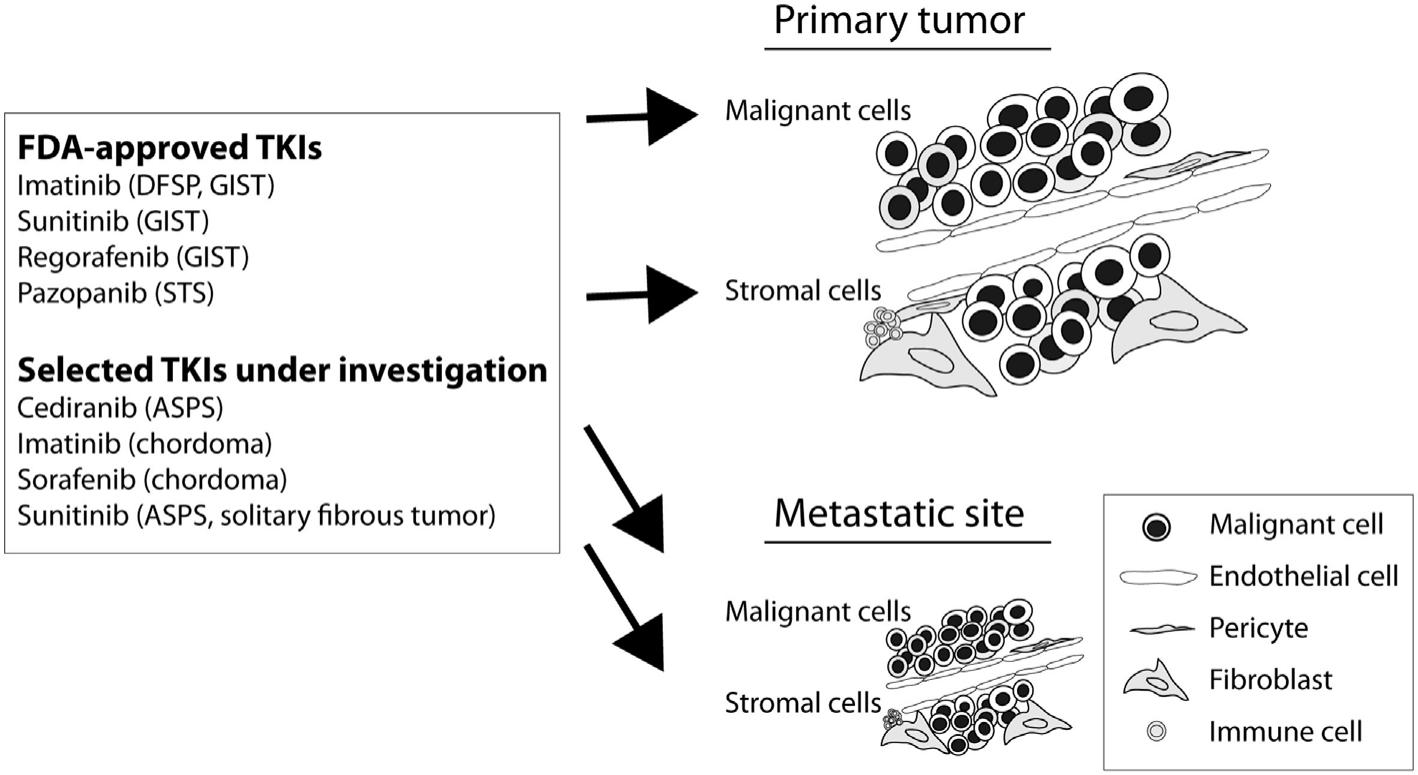
**Potential therapeutic effects of selected TKIs in sarcoma**. The biological mechanisms associated with patient response to TKIs in sarcoma are generally poorly characterized. Imatinib has mainly been described for its ability to target aberrant PDGFR signaling in dermatofibrosarcoma protuberans (DFSP) and KIT (or PDGFR) in advanced GIST. Sunitinib is approved for second-line treatment of GIST, after imatinib treatment failure, with molecular targets including VEGFR, PDGFR, FLT3, KIT, CSF1R, and RET. Regorafenib is approved for third-line treatment of GIST, after sunitinib treatment failure, with molecular targets including VEGFR, KIT, RET, FGFR, PDGFR, and RAF. Pazopanib targets VEGFR, PDGFR, KIT, and RAF and is approved for use in advanced STS. Cediranib and sunitinib are investigated for their ability to target VEGFR signaling in, e.g., alveolar soft part sarcoma (ASPS). Imatinib and sorafenib are investigated for therapeutic use in chordoma, a disease with reported PDGFR activity. Sorafenib has a target spectrum, including RAF, VEGFR, PDGFR, KIT, RET, and FLT3.

There is currently a great enthusiasm for targeted therapies as a novel complementary or alternative treatment method in cancer. A number of potential molecular targets have also been discussed in the context of translational sarcoma studies (48). So far, most phase II and phase III clinical trials where a therapeutic benefit has been confirmed include studies on tyrosine kinase inhibitors (TKIs) targeting VEGFRs, PDGFRs, and KIT. Given the mesenchymal origin of sarcomas, these agents are likely to target both tumor cells and infiltrating stromal cells in a context-dependent manner (Figure 1). For more details, the reader is referred elsewhere (36, 49). Selected agents with presumed microenvironmental effects are described below.

### Therapies with Antiangiogenic Effects

Angiogenesis was early predicted to be a common denominator for targeted therapy in a broad range of tumor types (17) and multiple studies have confirmed that antiangiogenic therapy causes starvation and reduced growth of experimental tumors. Accumulating evidence, however, suggest that the initial therapeutic benefit is followed by resistance mechanisms where the hypoxic and metabolic response to antiangiogenic therapy could worsen tumor aggressiveness (50).

One of the current antiangiogenic strategies is to alleviate hypoxia while improving tumor perfusion (51). Ideally, this approach increases response rates to radiotherapy, chemotherapy, and immunotherapy (5). Toxicity may however be a concern of some combination treatments. Of note is that the commonly targeted VEGFR pathway has not only been associated with induction of angiogenesis in STS but also with chemoresistance and regulatory T cell activity (33, 38).

The therapeutic efficacy of antiangiogenic therapy has been investigated in several sarcoma clinical trials. The TKI pazopanib was approved by the Food and Drug Administration (FDA) in 2012 for the use in advanced STS based on the results presented from the PALETTE phase III study (52). Anti-tumor activity has been demonstrated both in terms of sarcoma cell viability and inhibition of angiogenesis in a sarcoma cell line-dependent manner (53, 54).

Imatinib is another FDA-approved TKI with established ability to target KIT-mutated advanced GISTs. VEGF activity has, however, been described in GIST and imatinib can suppress GIST VEGF expression *in vitro* (55). Imatinib-responding GIST patients also display decreased serum VEGF levels. These antiangiogenic effects of imatinib, and other TKIs, have been reviewed by others (36). Only occasional responses to imatinib monotherapy have been observed in non-GIST sarcoma patients (56).

Sunitinib and regorafenib are both FDA-approved therapies for advanced GIST (second-, and third-line treatment, respectively) after failure to respond to imatinib. Their effects on tumor stroma, including angiogenesis, have not been clearly separated from the anti-proliferative effects on tumor cells (57–59).

### Therapies with Immune-Modulating Effects

Several cancer therapies in sarcoma may have direct or indirect effects on the immune system (38, 60, 61). Chemotherapy can, e.g., induce immunogenic cell death in tumors, block the immunosuppressive functions of myeloid-derived suppressive cells and likely lead to a more pronounced anti-tumor response. Trabectedin, approved by the European Medicines Agency (EMA) for second-line treatment of advanced STS, is an example of a chemotherapeutic agent, with the additional ability to induce apoptosis selectively in monocytes/macrophages (62).

The EMA approved the immunostimulant mifamurtide in 2009 for the use in high-grade non-metastatic osteosarcoma in combination with postoperative chemotherapy. The treatment was shown to significantly improve 6-year overall survival from 70 to 78% (63). Additional studies on the therapeutic efficacy are however warranted (2, 63, 64). Mifamurtide is reported to have its mechanism of action on macrophages and monocytes.

An illustrative example of a targeted therapy with immune-modulating side effects is imatinib treatment in advanced GIST, where an immunologic interferon-γ response has been associated with long-term survival (65). The antiangiogenic, anti-tumor, and immunostimulating roles of interferons have been reviewed elsewhere (66).

Denosumab is an FDA-approved monoclonal antibody directed against RANKL, which is expressed by the neoplastic cells in giant-cell tumor of bone (67). Osteoclasts, their precursors and reactive osteoclast-like giant cells express the receptor RANK. RANKL–RANK signaling contributes to osteoclast formation, osteolysis, and tumor growth. This type of tumor is often benign, but with unpredictable behavior.

### Targeted Therapies Under Investigation

Ongoing and future studies will reveal to what extent current targeted therapies under investigation have anti-tumor effects associated with the tumor microenvironment. Selected examples include the antiangiogenic TKIs sunitinib and cediranib with potential anti-tumor activity in ASPS, a malignancy associated with oncogenic MET signaling, pro-angiogenic factors, and inflammatory components (38, 68–70). Abundant VEGF expression has been confirmed. Sunitinib has also shown activity in solitary fibrous tumors (71). In the latter study, all cases were positive for PDGFRβ and/or VEGFR2.

Sorafenib is another widely used TKI with potential activity in subsets of sarcomas, either as mono- or combination therapy (72). Anti-tumor effects are believed to occur by several molecular mechanisms including inhibition of RAF, VEGFRs, PDGFRs, and KIT. Recently, the early results from a sorafenib phase II trial with locally advanced and metastatic chordoma patients were presented and compared with the results from two previous chordoma phase II trials with imatinib and lapatinib, respectively (73). Response rates were modest. Chordomas frequently express growth factor receptors, such as PDGFRs and EGFRs (74), and VEGF expression has been confirmed (75, 76).

An example of an antibody-based targeted therapy with promising activity is the use of olaratumab, an anti-PDGFRα monoclonal antibody, in combination with doxorubicin in advanced STS (abstract 10501, ASCO Annual meeting 2015). In a randomized phase Ib/II study, an improvement of 10.3 months in overall survival was achieved compared to doxorubicin alone (HR = 0.44, *p* = 0.0005).

It is yet too early to say whether novel immune-modulating therapies will be of therapeutic value in sarcoma. Immune checkpoint inhibitors have emerged as a promising therapy in other tumor types and are currently being tested in sarcoma. T-cell receptor-based gene therapy directed against tumor-specific antigens is another type of treatment with promising activity in synovial sarcoma (77).

## Concluding Remarks

Treatment options are today limited for many sarcoma patients. The infiltrative growth pattern of many sarcomas makes complete tumor resection with negative margins difficult. Distant metastases are often present already at diagnosis. Further preclinical and clinical studies are clearly needed to identify novel therapeutic targets.

The future directions of sarcoma diagnosis, therapy and follow-up are likely to rely more on tumor-specific biology. Careful monitoring of individual tumor genetics and gene/protein expression patterns is predicted to be essential. Therapy-adapted screening methods and standard criteria for tumor response assessment beyond the response evaluation criteria in solid tumors (RECIST) need to be further developed. Useful biomarkers, stromal components, and microenvironmental targets of importance for disease progression largely remain to be identified within the new era of personalized medicine.

## Author Contributions

ME wrote the manuscript, edited, and approved the final version to be published. OL critically revised the content of the work and approved the final version to be published.

## Conflict of Interest Statement

The authors declare that the research was conducted in the absence of any commercial or financial relationships that could be construed as a potential conflict of interest.

## Abbreviations

ASPS, alveolar soft part sarcoma; CSF1R, colony stimulating factor 1 receptor; DFSP, dermatofibrosarcoma protuberans; EGFR, epidermal growth factor receptor; EMA, European Medicines Agency; EWS/FLI, Ewing sarcoma breakpoint region 1; FDA, Food and Drug Administration; FGFR, fibroblast growth factor receptor; FLT3, fms-related tyrosine kinase 3; GIST, gastrointestinal tumor; KIT, v-kit Hardy-Zuckerman 4 feline sarcoma viral oncogene homolog; MET, hepatocyte growth factor receptor; PAX3-FOXO1, paired box 3-forkhead box O1; PDGFR, platelet-derived growth factor receptor; RAF, zinc fingers and homeoboxes 2; RANKL, tumor necrosis factor (ligand) superfamily, member 11; RECIST, response evaluation criteria in solid tumors; RET, ret proto-oncogene; STS, soft tissue sarcoma; TKI, tyrosine kinase inhibitor; VEGFR, vascular endothelial growth factor receptor; vWF, von Willebrand factor; WHO, World Health Organization.
